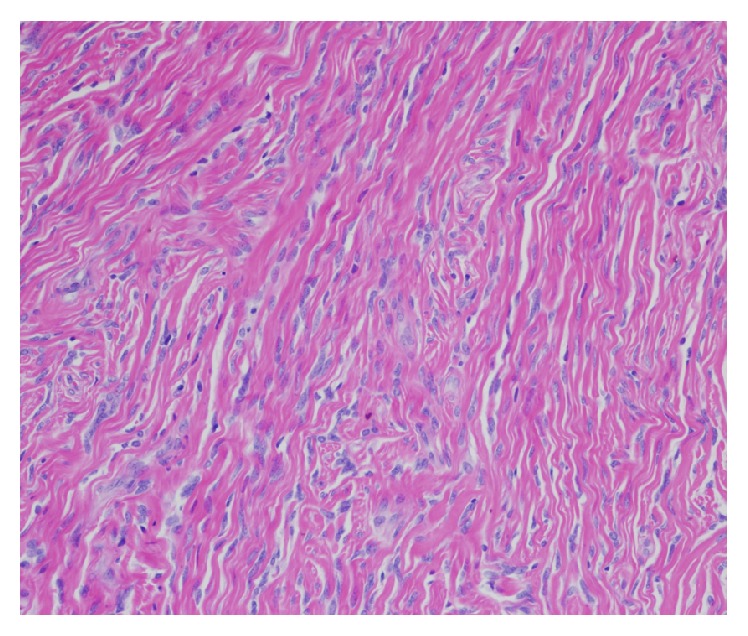# Urethral Solitary Fibrous Tumor: A Rare Pathologic Diagnosis of a Periurethral Mass

**DOI:** 10.1155/2016/2798079

**Published:** 2016-08-08

**Authors:** Gaby N. Moawad, Elias D. Abi Khalil, Cheryl Silverbrook, Stephanie Barak, Alice Semerjian, Michael Phillips

**Affiliations:** ^1^Department of Obstetrics and Gynecology, The George Washington University, Washington, DC 20052, USA; ^2^Department of Pathology, The George Washington University, Washington, DC 20052, USA; ^3^Department of Urology, The George Washington University, Washington, DC 20052, USA

## Abstract

Solitary fibrous tumors (SFTs) may occur at any site in the body. SFTs can only be conclusively diagnosed based on histopathologic and immunohistochemical characteristics of the tumor. The presence of SFTs in the abdomen and pelvis is extremely rare. To our knowledge no cases of urethral solitary fibrous tumor in the literature have been reported so far. We present a case of a solitary fibrous tumor arising from the urethra in a twenty-three-year-old female presenting with vaginal mass.

## 1. Case Presentation

A 23-year-old sexually active female with no significant medical or surgical history initially presented to colorectal surgery for evaluation of a potential perianal mass. The patient reports having limited medical care and no recent gynecological visits. Three years prior to presentation, the patient first noticed a small growth on her perineum. Over time this growth had increased in size, but it had not interfered with any of her activities. She was still able to have sex and urinate without difficulty. The patient had no reported history of sexually transmitted infections. She denied significant pain, discharge, or bleeding from this mass.

**Figure 1:**
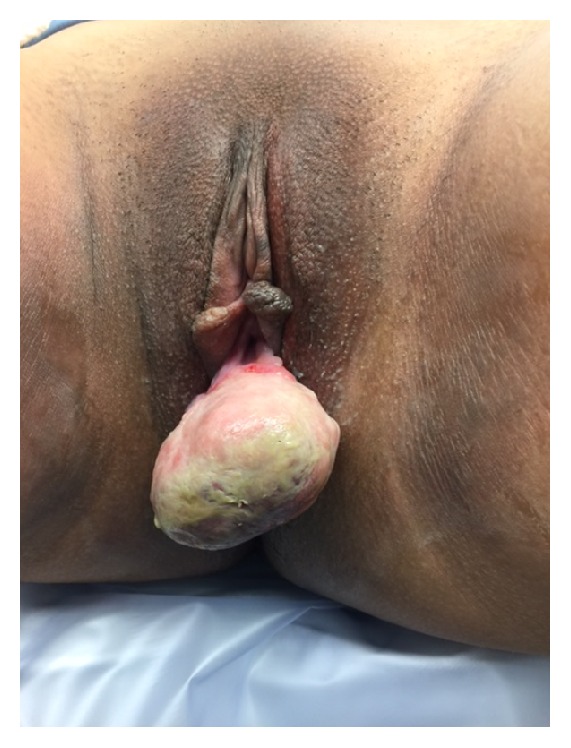


**Figure 2:**
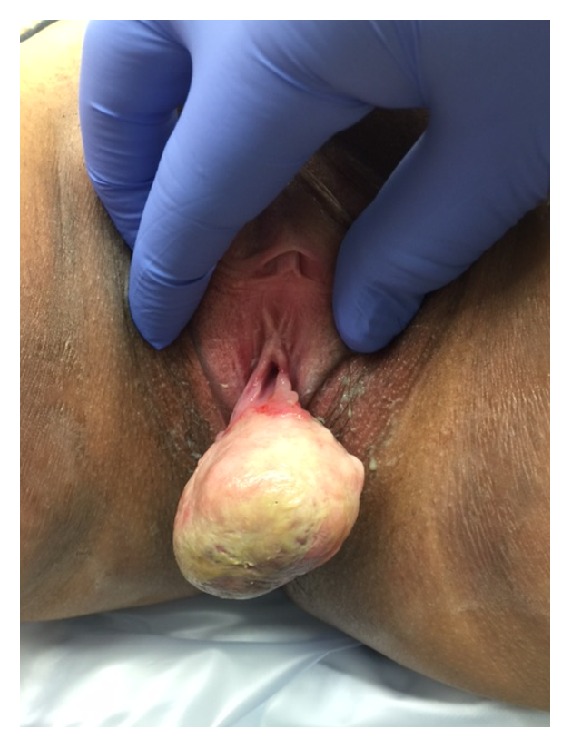


During the initial physical examination by the colorectal surgeon it was determined that this lesion was not arising from the perianal region but was in fact of periurethral or anterior vaginal wall origin (Figure 1). The patient was referred to urology for further evaluation and discussion of surgical planning. Physical examination by the urologist revealed a 5 cm × 6 cm firm, nodular, mobile mass attached to the posterior lip of the urethra (Figure 2). Further examination of the urethra did not reveal any additional masses or extensions. No preoperative imaging was performed.

The patient was scheduled for an examination under anesthesia, cystoscopy, and planned vaginal excision of the mass from the posterior urethral edge. Further imaging with ultrasound, CT scan, or MRI was deferred pending final pathologic findings.

**Figure 3:**
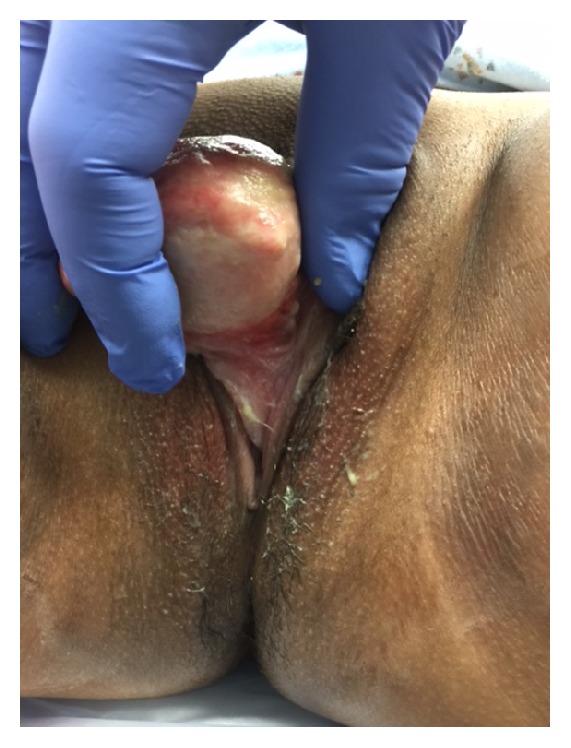


**Figure 4:**
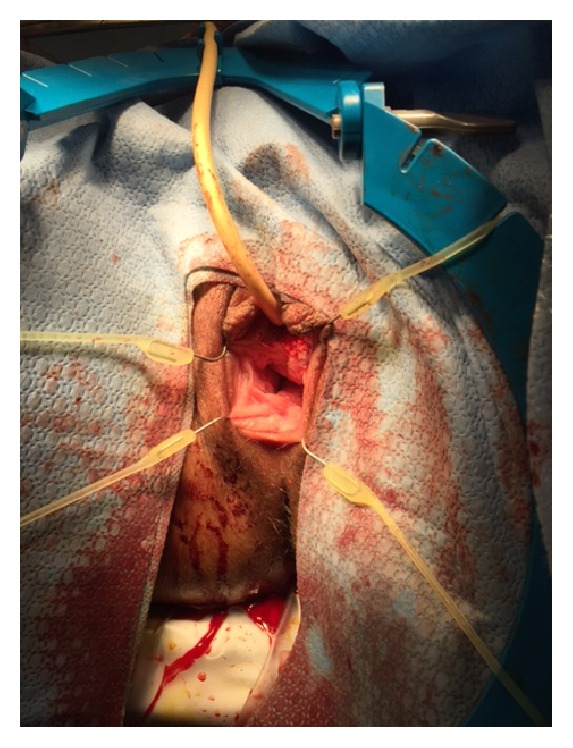


At the time of a surgery, cystourethroscopy showed no evidence of urethral involvement with the mass. The bladder neck and bladder itself were noted to be unremarkable with no evidence of tumor, stones, or diverticulum. After completion of cystourethroscopy, a foley catheter was placed, and the mass was placed on traction. The mass was noted to have a broad base spanning from the 4 o'clock to 8 o'clock position encompassing the anterior vagina and posterior lip of the urethra (Figure 3). The base was transected using electrocautery. At time of transection, it was noted that the base was very vascular and electrocoagulation was used to achieve hemostasis. The urethra was everted and oversewn to the cut edge of the vaginal tissue in an interrupted fashion with 4-0 chromic (Figure 4).

A frozen section was performed intraoperatively. The pathologist noted benign cytological features with no obvious source. The cut margin appeared free of any involvement. At the end of the case, the patient was transferred to the recovery room in stable condition with foley catheter and vaginal packing in place. Both the foley and vaginal packing were removed prior to the patient being discharged home.

**Figure 5:**
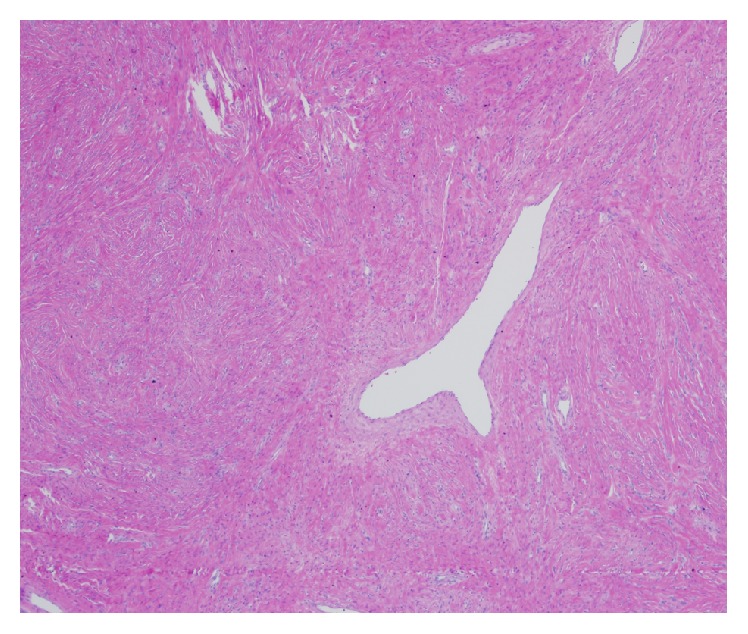


That patient presented for a two-week postoperative appointment with urologist with no acute complaints. She was recovering well from her procedure. At her follow-up appointment final pathology results were as follows:

 The mass measured 5.6 cm in maximal dimension. The mass was polypoid and pedunculated with a smooth and glistening external surface. A section of skin was recognizable. The cut section was homogenous with a tan/white color. The microscopic sections revealed a well circumscribed, unencapsulated mesenchymal neoplasm composed of bland spindle cells with elongated nuclei and indistinct cytoplasm. The cells were arranged randomly with hyper- and hypocellular areas (patternless pattern), in a collagenized matrix with variably prominent, often staghorn-shaped blood vessels (Figure 5). Focal chronic inflammation and myxoid changes were present. Mitotic figures, cellular atypia, or necrosis was not identified (Figure 6). There was surface ulceration with associated reactive changes and dense collagen, surrounding the lesion.

Immunohistochemically, the spindle cells were positive for CD34, bcl2, and CD99 (focally) and negative for S-100, desmin, and smooth muscle actin. These findings are consistent with a solitary fibrous tumor of the urethra.

## 2. Discussion

Periurethral masses are rare, occurring in only 3-4% of all patients [1]. It can be difficult to distinguish urethral, periurethral, and anterior vaginal wall masses by physical examination. Anterior vaginal wall masses are even less common than those located periurethrally, with a prevalence of approximately 1% [1]. The differential diagnosis for a patient who presents with a concern for a periurethral versus an anterior vaginal wall mass is broad but should include the following conditions: urethral caruncle, periurethral cyst (Skene's duct cyst), urethral prolapse, ectopic ureterocele, urethral diverticulum, vaginal wall cyst of embryonic origin (Müllerian and Gartner's duct cysts), leiomyoma, malignant neoplasm of urethral or vaginal origin, and solitary fibrous tumor [1].

Solitary fibrous tumors were first described in 1931. These tumors are of fibroblastic or myofibroblastic origin [2]. Solitary fibrous tumors (SFTs) may occur at any site in the body but are most often localized in the pleura [2]. The presence of SFTs in the abdomen and pelvis is extremely rare.

Extrapleural SFTs are observed in middle aged adults, typically between 20 and 70 years of age, with both sexes being affected equally [3]. Reports in the literature have shown that extrapleural SFTs can range in size from 1 cm to 25 cm [3]. These lesions tend to be tan-colored, rubbery masses that are often tethered by a pedicle and are partially or totally encapsulated [3].

SFTs can only be conclusively diagnosed based on histopathologic and immunohistochemical characteristics of the tumor. SFTs are classically described as having a “patternless pattern.” Microscopic examination shows spindle cells dispersed among dense collagen fibers in a storiform arrangement [4]. Tumor cells are CD34, CD99, and bcl2 positive, while the S-100 protein, actin, and desmin are negative [4].

Most of these tumors are asymptomatic unless they grow to cause compressive or obstructive symptoms. In our case, the patient was asymptomatic but the size of the tumor grew to be bothersome to her daily activities. Rarely, these tumors can be associated with Doege-Potter syndrome. This is a syndrome in which the tumor is associated with hypoglycemia, which occurs with less than 5% of all SFTs [5]. The cause of hypoglycemia is related to insulin-like growth factors produced by these tumors [5].

SFTs are reported to be malignant in 10–15% of cases [6]. The rates of malignancy may be as high as 20% in pleural SFTs, with lower rates in extrapleural locations. Due to the potential risk of malignancy, the treatment of choice for these tumors is surgery with local excision and clean margins. Positive margins account for up to 40% of local recurrences and 75% of metastases [7]. Most recurrences occur in the first 24 months after resection [7].

Surgical removal of a periurethral or urethral mass can result in surgical complications. One of the most concerning complications results from scarring of the urethra. This can cause a urethral stricture resulting in bladder outlet obstruction. Bladder outlet obstruction occurs in 2–29% of all women [8]. This obstruction presents as weak stream, dribbling, recurrent urinary tract infections, and detrusor overactivity [8]. Urethral strictures account for 4–18% of all women with bladder outlet obstructions [8]. If a woman develops this complication postoperatively it can be treated with urethral dilation or urethroplasty.

A PubMed search did not yield any reports of urethral solitary fibrous tumor in the literature. There have been several case reports of SFTs arising in other areas of the urogenital tract and female genital tract including the kidney, bladder, vulva, and vagina. Although uncommon, solitary fibrous tumors should be in the differential diagnosis for any soft tissue mass arising from the urogenital tract.

## Figures and Tables

**Figure 6:**